# Leveraging large-scale datasets and single cell omics data to develop a polygenic score for cisplatin-induced ototoxicity

**DOI:** 10.1186/s40246-024-00679-5

**Published:** 2024-10-08

**Authors:** Deanne Nixie R. Miao, MacKenzie A. P. Wilke, John Pham, Feryal Ladha, Mansumeet Singh, Janilyn Arsenio, Emilia Luca, Alain Dabdoub, Wejian Yang, Jun J. Yang, Britt I. Drögemöller

**Affiliations:** 1 Department of Biochemistry and Medical Genetics, Rady Faculty of Health Sciences, University of Manitoba, Winnipeg, MB Canada; 2 Department of Internal Medicine, Max Rady College of Medicine, University of Manitoba, Winnipeg, MB Canada; 3 Sunnybrook Research Institute, Toronto, ON Canada; 4 Department of Pharmacy and Pharmaceutical Sciences, St. Jude Children’s Research Hospital, Memphis, TN 38105 USA; 5 Department of Oncology, St. Jude Children’s Research Hospital, Memphis, TN 38105 USA; 6 Hematological Malignancies Program, St. Jude Children’s Research Hospital, Memphis, TN 38105 USA; 7 CancerCare Manitoba Research Institute, Winnipeg, MB Canada; 8 Children’s Hospital Research Institute of Manitoba, Winnipeg, MB Canada; 9 Centre of Aging, University of Manitoba, Winnipeg, MB Canada

**Keywords:** Cisplatin-induced ototoxicity, Pharmacogenomics, Polygenic scores, Single-nuclei RNA-sequencing

## Abstract

**Background:**

Cisplatin-induced ototoxicity (CIO), characterized by irreversible and progressive bilateral hearing loss, is a prevalent adverse effect of cisplatin chemotherapy. Alongside clinical risk factors, genetic variants contribute to CIO and genome-wide association studies (GWAS) have highlighted the polygenicity of this adverse drug reaction. Polygenic scores (PGS), which integrate information from multiple genetic variants across the genome, offer a promising tool for the identification of individuals who are at higher risk for CIO. Integrating large-scale hearing loss GWAS data with single cell omics data holds potential to overcome limitations related to small sample sizes associated with CIO studies, enabling the creation of PGSs to predict CIO risk.

**Results:**

We utilized a large-scale hearing loss GWAS and murine inner ear single nuclei RNA-sequencing (snRNA-seq) data to develop two polygenic scores: a hearing loss PGS (PGS_HL_) and a biologically informed PGS for CIO (PGS_CIO_). The PGS_CIO_ included only variants which mapped to genes that were differentially expressed within cochlear cells that showed differential abundance in the murine snRNA-seq data post-cisplatin treatment. Evaluation of the association of these PGSs with CIO in our target CIO cohort revealed that PGS_CIO_ demonstrated superior performance (*P* = 5.54 × 10^− 5^) relative to PGS_HL_ (*P* = 2.93 × 10^− 3^). PGS_CIO_ was also associated with CIO in our test cohort (*P* = 0.04), while the PGS_HL_ did not show a significant association with CIO (*P* = 0.52).

**Conclusion:**

This study developed the first PGS for CIO using a large-scale hearing loss dataset and a biologically informed filter generated from cisplatin-treated murine inner ear snRNA-seq data. This innovative approach offers new avenues for developing PGSs for pharmacogenomic traits, which could contribute to the implementation of tailored therapeutic interventions. Further, our approach facilitated the identification of specific cochlear cells that may play critical roles in CIO. These novel insights will guide future research aimed at developing targeted therapeutic strategies to prevent CIO.

**Supplementary Information:**

The online version contains supplementary material available at 10.1186/s40246-024-00679-5.

## Background

Cisplatin is an effective platinum-based chemotherapeutic that is used to treat a spectrum of malignancies, including leukemias, lymphomas, sarcomas, as well as breast, testicular, ovarian, head and neck, and cervical cancers [1]. The effectiveness of this treatment is highlighted by the fact that the introduction of cisplatin into testicular cancer treatment protocols in 1978 led to a 9-fold increase in survival rates for these patients [2, 3]. Unfortunately, cisplatin treatments are also accompanied by the occurrence of adverse drug reactions (ADRs), which can significantly diminish patients’ quality of life [4, 5]. One such example is cisplatin-induced ototoxicity (CIO), a common ADR characterized by permanent, progressive, and bilateral hearing loss [6]. The exact mechanisms underlying the development of CIO are still not completely understood, but several pathways have been implicated [5]. This includes damage to cochlear cells through the cytotoxic effects of cisplatin, damage to nuclear and mitochondrial DNA [7, 8], and the activation of apoptosis [9] through direct and indirect means [10–12].

The incidence of ototoxicity after cisplatin treatment, which can be up to 80% for some cancers [13], adds to the challenges and burdens already faced by cancer patients and survivors. Further, the development of hearing loss is associated with adverse consequences throughout these individuals’ lives, including challenges in speech and language development for young children, as well as the potential for increased social isolation, or depression [14]. As sodium thiosulfate has recently been approved by the Food and Drug Administration (FDA) to reduce the risk of CIO in pediatric patients with non-malignant cancers [15], CIO risk prediction models could help to prioritize which individuals would benefit most from these otoprotectants. Several risk factors for CIO have been identified, including age, with pediatric and older adults at higher risk [5], high cisplatin administration schedules [16], and cranial irradiation, which is considered the most significant clinical risk factor for CIO [17]. As a result, current predictive models for CIO have focused on using clinical risk factors to predict risk of CIO [18, 19].

While clinical factors play an important role in risk of CIO, they do not account for all the variability observed for this ADR. In line with this, heritability studies suggest that genetic variation also plays an important role in CIO, with up to 47% of the variability in the occurrence of CIO attributed to genetics [20]. Notably, genome-wide association studies (GWAS) have identified an increasing number of genetic variants that contribute to CIO risk [17, 21, 22]. Unfortunately, due to the polygenic nature of CIO, individually, these genetic variants offer only partial insights into overall genetic risk for CIO [23]. This highlights the need for polygenic scores (PGS), which integrate risk information from multiple genetic variants across the genome to create a single risk score for each individual. However, accurately generating these scores for CIO remains challenging due to relatively small cohorts, resulting in inaccurate effect size estimates for risk variants [24]. Therefore, innovative approaches are required to overcome these challenges and enhance the accuracy of PGSs.

To overcome these limitations in this study, we integrated data from a large-scale multi-trait analysis of GWAS (MTAG) of hearing loss [25] with cisplatin-treated murine inner ear single nuclei RNA sequencing (snRNA-seq) data. Due to the genetic overlap between hearing loss phenotypes and CIO [26], we used MTAG data to increase our power to uncover variants that play a role in several diverse hearing traits. Further, given the potential tissue-specific effects of cisplatin on gene expression, the generation of inner ear snRNA-seq data obtained from cisplatin-treated mice allowed for the incorporation of a biologically informed refinement filter to enhance the relevance of our initial PGS (i.e., PGS_HL_) to CIO. By harnessing these unique data, we developed a refined PGS (i.e., PGS_CIO_) that was associated with CIO in two independent cohorts, providing further insights into the genetic factors that influence susceptibility to CIO.

## Methods

### Study cohorts

#### Base cohort

Given the observed overlap in the genetic architecture between hearing loss and CIO [26] we obtained summary statistics from a previously published MTAG of four genetically correlated, heritable and polygenic hearing loss traits from the UK Biobank (UKB) [25]. These data were used as a base cohort to develop a hearing loss PGS that would be of relevance to diverse hearing loss traits. The four self-reported hearing traits included in this MTAG were hearing difficulty with background noise (cases: *n* = 134,141; controls: *n* = 219,842), hearing difficulty (cases: *n* = 90,710; controls: *n* = 255,925), hearing aid user (cases: *n* = 10,942; controls: *n* = 208,416), and tinnitus (cases: *n* = 7,739; controls: *n* = 110,142). The use of this large-scale MTAG dataset allowed us to circumvent sample size limitations associated with current CIO GWAS, providing more accurate effect size estimates for variants that are associated across diverse hearing loss phenotypes [24].

#### Target cohort

To investigate the association between the PGSs and CIO, GWAS summary statistics from the PanCareLIFE (PCL) cohort were obtained. This cohort is described in detail elsewhere [27], but consists of 390 pediatric cancer patients of European (92%), and non-European ancestry (8%) (African-American/African-Caribbean and Latin American) who underwent cisplatin treatment, but were not subjected to cranial radiation therapy. Cases and controls were defined by the Muenster grading criteria, with 168 individuals exhibiting CIO (Muenster grade > 2b), while 222 individuals showed no or minimal ototoxicity (Muenster grade 0-2a). Genotyping was performed using the Illumina Infinium Global Screening Array. Quality control (QC) and imputation, using the Michigan Imputation Server and the Haplotype Reference Consortium reference (HRC r1.1), were performed as previously described [27].

#### Test cohort

To validate the association between the PGSs and CIO, individual-level genotype and clinical data from two St. Jude’s Children Research Hospital Medulloblastoma (SJMB) cohorts, the SJMB96 cohort consisting of 31 children enrolled between 1996 and 2003 and the SJMB03 cohort consisting of 207 children enrolled between 2003 and 2012, were used. All individuals enrolled in both the SJMB96 and SJMB03 cohorts underwent craniospinal irradiation (CSI) and cisplatin treatment. Further, cases and controls were defined by the Chang grading criteria, with 61% of individuals exhibiting CIO (Chang grade$\:>$ 0), while 39% of individuals showed no ototoxicity (Chang grade 0). Genotyping of these samples was performed using the Illumina HumanOmni2.5+HumanExome BeadChip, as previously described [17].

### Generation of mouse inner ear single-nuclei RNA-sequencing data

Intraperitoneal injections of 3.0 mg/kg cisplatin or saline were administered to postnatal day-6 (P6) CBA/CaJ mice in the control (*n* = 6) and treatment groups (*n* = 6), respectively. This dosing schedule has been shown to produce a clinically relevant mouse model for CIO [28]. P6 mice were chosen for the absence of ossification in the cochlear cartilaginous membrane, facilitating optimal dissection of intact cochlea samples and eliminating the need to include decalcification processes which may alter gene expression profiles, thereby enhancing compatibility with snRNA-seq. snRNA-seq was used as these data form part of a larger multiome dataset, which requires nuclei for ATAC sequencing. Four hours post-treatment, corresponding to the timepoint when cisplatin-induced gene expression changes were observed in other organs in mice [29], mice were euthanized via decapitation and their cochleae were micro-dissected as previously described [30]. After dissection, the cochleae were flash-frozen in liquid nitrogen for 45 s and stored in a liquid nitrogen tank. Single nuclei were isolated from pooled treatment and control cochlea samples using the 10X Genomics Chromium Nuclei Isolation Kit, according to the manufacturer’s protocol, modified to include only one round of washing to allow for maximum nuclei yield. Before single-cell library preparation, the concentration and viability of the nuclei were assessed using AO/PI staining and the CellDrop automated cell counter. Libraries were prepared using the Single Cell GEX & Fixed RNA Profiling Kit and a local Chromium Controller, according to the manufacturer’s protocol. These libraries were frozen and sent for sequencing with a target depth of 50 million reads/sample on the NovaSeq X Plus Sequencing System at Princess Margaret Genomics Sequencing Centre. Technical replicates were performed for each sample by preparing two separate libraries from the same nuclei suspension across two channels on the Chromium microfluidic device.

After sequencing, data processing was carried out, as detailed in Supplementary Fig. 1. Alignment of reads was performed using CellRanger, followed by QC checks using FastQC. Further QC was performed to correct for ambient RNA using SoupX, filtering low-quality cells using Seurat, and doublet removal using scDblFinder. Low-quality cells were identified based on their mitochondrial percentages deviating more than 3 median absolute deviations (MADs), as well as cells with log10-transformed gene counts and UMI counts exceeding ±3 MADs. The data was normalized and scaled using Seurat’s *NormalizeData* and *ScaleData* functions. Feature selection identified informative genes, followed by principal component analyses (PCA) and cell clustering. Uniform Manifold and Approximation Projection (UMAP) was then used to visualize the data, and clusters were annotated using Seurat’s data transfer method based on previously published murine cochlear data [31].

### Construction of a hearing loss PGS (PGS_HL_)

MTAG summary statistics were harmonized using the S-PrediXcan harmonization pipeline [32] to ensure consistent formatting and compatibility across different datasets. These harmonized summary statistics, along with the GCTB sparse shrunk linkage disequilibrium (LD) reference panel consisting of 2.8 million common variants from the UKB, were used as inputs for SBayesR [33] to construct a PGS for hearing loss using Bayesian-based approaches. We used SBayesR default parameters with the “—robust” option to address potential differences in SNP effects across traits in the MTAG dataset [33]. Ambiguous variants (i.e., C/G, G/C, A/T and T/A variants) were excluded from downstream analyses.

### Refinement of PGS_HL_ to increase the relevance of the score to CIO (PGS_CIO_)

**Fig. 1:**
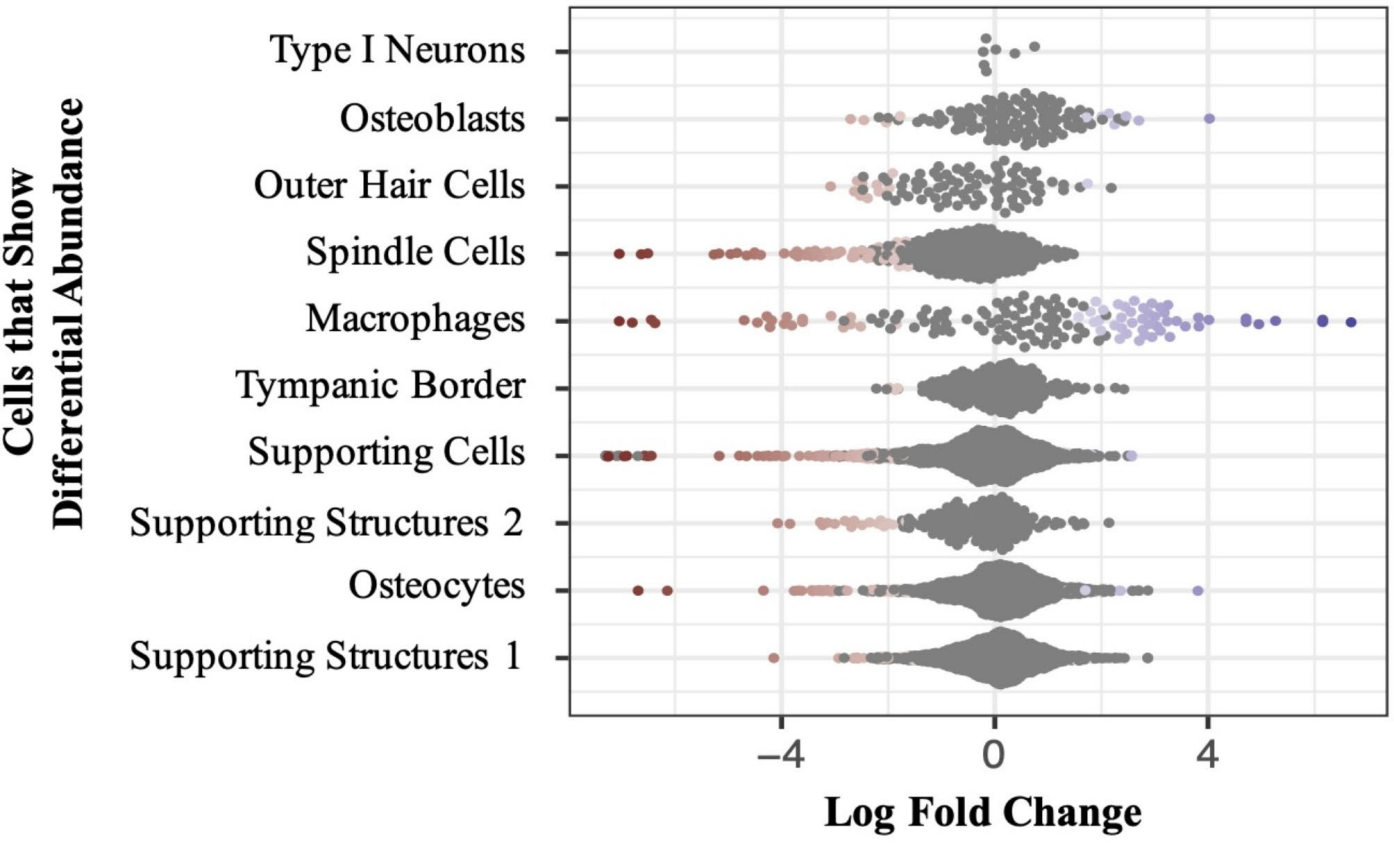
**Beeswarm plot illustrating cochlear cells showing differential abundance four hours post-cisplatin treatment vs. saline-treated samples.** Each dot represents a neighborhood of cells. Red indicates decreased abundance and blue indicates increased abundance of cells treated with cisplatin relative to saline-treated controls

To enhance the relevance of PGS_HL_ to CIO [24], a biologically informed pharmacogenomics filtering strategy was applied. To do this, analyses with MILO-R [34] were performed to identify cochlear cell types that exhibited differential abundance post-cisplatin treatment, when compared to saline-treated samples (LogFC<=-3, SpatialFDR < 0.1). Genes that were differentially expressed in these cells were identified using DESeq2 [35] (*P*_adj_<0.05). Next, Ensembl’s Variant Effect Predictor v.108.1 was used, with default settings, to map genetic variants included in the PGS_HL_ to human Ensembl gene IDs. Corresponding mouse orthologues were identified using the R package, babelgene. These data were then used to filter the PGS_HL_ to include only variants mapping to murine genes that were differentially expressed within cochlear cells that showed differential abundance in the snRNA-seq data post-cisplatin treatment (Fig. 1). This filtering strategy resulted in the generation of a biologically relevant PGS (PGS_CIO_) for downstream analyses, with effect size weights of variants included in this score derived from the base cohort.

### Testing the association between PGS_HL_/PGS_CIO_ and CIO in the target cohort

The association between PGS_HL_/PGS_CIO_ and CIO was evaluated using the CIO GWAS summary statistics from the PCL cohort. To enable comparison between datasets, the summary statistics were harmonized using the S-PrediXcan pipeline. These harmonized summary statistics were used to assess the associations between the PGS_HL_/PGS_CIO_ and CIO using ReACt [36]. The use of this tool circumvented the need to obtain individual-level data, thereby streamlining these analyses [37]. By reconstructing allelic frequencies [36] from the summary statistics obtained from the target (PCL) cohort, ReACt was used to calculate the mean PGS in cases and controls and test for significant differences between CIO cases and controls using a two-sample *t*-test [36]. *P* < 0.05 was considered statistically significant.

### Validating the association between PGS_HL_/PGS_CIO_ and CIO in the test cohort

Genomic QC was performed using PLINK v1.09 (Supplementary Fig. 2). Variants with missing data exceeding 5% and deviating from Hardy Weinberg Equilibrium (*P* < 1.0 × 10^− 4^) were removed, along with individuals exhibiting missing data exceeding 5%, related individuals (PI hat > 0.2), and those with mismatched sex. Imputation was performed using the TOPMed Imputation Server and the TOPMed r3 reference panel. Following imputation, all variants with *R*^*2*^ > 0.95 were included in the data to ensure the inclusion of high-quality imputed genotypes. After pruning for LD, and removing variants with minor allele frequencies < 0.01, PCA was performed using EIGENSOFT v5. Principal component clustering revealed that 69% of patients were of European descent.

Using PLINK v1.09, PGSs were generated for each individual in the test cohort for both the PGS_HL_ and the PGS_CIO_. After scoring individuals, the distribution of PGSs were evaluated for normality using the Shapiro test. Deviation from normality was indicated by *P* < 0.05. To assess the association between PGS_HL_/PGS_CIO_ and CIO, logistic regression was performed using the geneticriskR package [38] in R, including age at diagnosis, protocol (SJMB96 or SJMB03) [17], and the first ten ancestral principal components as covariates. These covariates were selected based on their previously reported significant associations with CIO [17]. Further, given the established role of CSI in ototoxicity [39], we incorporated an interaction term between CSI dose (< 25 Gy or ≥25 Gy) and PGS in our model. The ability of significant PGSs to distinguish cases (individuals with CIO) and controls (individuals without CIO) was evaluated using receiver operating characteristic (ROC) curves and area under the curve (AUC) values. Lastly, the positive predictive value (PPV) and negative predictive value (NPV) were calculated to quantify the predictive accuracy of the PGS model. *P* < 0.05 was considered significant in all analyses.

## Results

### Generation of snRNA-seq data

To allow for biologically informed filtering of the PGS_HL_, we generated snRNA-seq from the inner ears of cisplatin-treated mice. After QC, we observed fewer cells in the cisplatin treatment group (replicate 1: *n* = 10,407 cells, replicate 2: *n* = 9,822) compared to the saline treatment control group (replicate 1: *n* = 13,212 cells, replicate 2: *n* = 12,724 cells). Closer investigation of the specific cells that are impacted by cisplatin treatment revealed that there was a decrease in type I neurons, osteoblasts, outer hair cells (OHC), spindle cells, macrophages, tympanic border cells, supporting cells, supporting structures 1 and 2, and osteocytes post-cisplatin treatment (MILO-R: LogFC<=-3, SpatialFDR < 0.1). Notably, macrophages were the only population of cells that demonstrated a significant increase in response to cisplatin treatment. Across the cochlear cell types that showed differential abundance, 159 differentially expressed genes (DEGs) were observed (DESeq2: *P*_adj_<0.05) (Supplementary Fig. 3).

### Evaluation of the association between PGS_HL_/PGS_CIO_ and CIO in the target cohort

A total of *n* = 2,370,365 non-ambiguous variants were included in PGS_HL_. Subsequent application of our refinement filter to PGS_HL_ yielded PGS_CIO_, which contained *n* = 138,670 variants mapping to DEGs within cochlear cells that showed differential abundance. ReACt was then used to determine whether there was a significant association between the PGSs and CIO in the target PCL cohort. While the PGS_HL_ was significantly associated with CIO in this cohort (*P* = 2.93 × 10^− 3^, *R*^*2*^ = 0.023), the PGS_CIO_ was more significantly associated with CIO (*P* = 5.54 × 10^− 5^, *R*^*2*^ = 0.041).

### Evaluation of the association between PGS_HL_/PGS_CIO_ and CIO in the test cohort

Investigation of the distribution of PGS_HL_ and PGS_CIO_ confirmed that these scores were normally distributed in the SJMB cohort (PGS_HL_: *P* = 0.72; PGS_CIO_: *P* = 0.96) (Supplementary Fig. 4). Examination of the association between PGS_HL_/PGS_CIO_ and CIO revealed that PGS_HL_ was not significantly associated with CIO in the test cohort (*P* = 0.52, Nagelkerke *R*^*2*^ = 0.006), while PGS_CIO_ demonstrated a significant association (*P* = 0.043, Nagelkerke *R*^2^ = 0.024). To establish baseline comparisons, we evaluated the independent predictive performance of a model including (i) only the PGS_CIO_, (ii) only clinical covariates (i.e., age at diagnosis, treatment protocol, CSI, and ancestral principal components), and (iii) the combined model, which incorporated both the PGS_CIO_ and the relevant clinical covariates, including the interaction between CSI and PGS_CIO_. The model which included only PGS_CIO_ yielded an AUC of 0.576 (95% CI: 0.503–0.650). Addition of PGS_CIO_ to the model that included the relevant clinical covariates did not substantially increase the predictive performance of the clinical model (AUC of 0.732, 95% CI: 0.668–0.797 vs. AUC of 0.714, 95% CI: 0.648–0.78) (Supplementary Fig. 5). Examination of the PPV (81%) and NPV (48%) revealed that the predictive model, which includes both clinical and genetic variables, correctly identifies 81% of true positive CIO cases and 48% of true negative CIO cases.

## Discussion

PGSs have shown considerable promise in various clinical contexts. For example, coronary artery disease (CAD) PGSs have identified a significantly larger number of at-risk individuals compared to methods relying on rare mutations [24, 40]. In addition, CAD PGSs have guided treatment decisions, such as the use of statins for patients classified as high-risk [24, 41]. Building on these successes, this is the first study to develop a hearing loss PGS that is specifically aimed at predicting CIO. By employing innovative approaches that integrate large-scale hearing loss GWAS data with cutting-edge snRNA-seq data, this study created a PGS that was significantly associated with CIO in two independent cohorts. This work has shown that the incorporation of omics datasets into PGS development pipelines can open new avenues for considering biological pathways underlying pharmacogenomic traits. Collectively, these novel methodologies offer a new approach for the development of pharmacogenomic PGSs.

### The use of large-scale databases and summary statistics to overcome sample size and resource limitations associated with the development of PGS for CIO

As reviewed in detail by Johnson et al., previous studies have demonstrated the value of using large-scale GWAS of related phenotypes (e.g. schizophrenia GWAS) to create PGSs that can be used to predict pharmacogenomic traits (antipsychotic response) [42]. Given the observed overlap in the genetic architecture of hearing loss and CIO [26], we decided to build upon these approaches by using MTAG summary statistics from the UKB cohort. These summary statistics offer unique benefits as they provide information relating to associations across four genetically correlated hearing loss traits [43], thereby allowing for the identification of genetic variants of relevance across diverse hearing phenotypes. In addition, MTAG leverages the shared genetic architecture among these traits to refine effect size estimates, thereby enhancing our ability to detect associations, with these approaches being reported to increase the variance explained by the PGSs by as much as 25% [43]. Leveraging this approach, our study identified a significant association between the PGS_HL_ and CIO in the PCL cohort. Further the use of summary statistics for both the base and target cohorts represents a cost- and resource-effective method for the preliminary evaluation of the performance of PGSs. Given the challenges related to data-sharing practices in pharmacogenomics research [37], this method provides researchers with a unique opportunity to assess the feasibility and robustness of PGSs for future investigation, even when individual-level data is not readily available.

### The use of snRNA-seq data to increase the relevance of a hearing loss PGS to CIO

The use of large-scale GWAS data from related phenotypes has been shown to be a useful approach for the development of pharmacogenomic PGS [42]. However, not all variants uncovered from these GWASs are likely to be relevant to their drug-induced counterpart phenotypes. Therefore, to enhance the relevance of the PGS_HL_ to CIO, we generated murine inner ear snRNA-seq data obtained from cisplatin-treated mice. By using snRNA-seq data to apply a pharmacologically relevant filter to our PGS_HL_, we were able to selectively include variants from the PGS_HL_ that map to DEGs within cochlear cells that showed differential abundance. By enhancing the biological relevance of PGS_HL_ to CIO, we developed a PGS (PGS_CIO_) with improved predictive capacity. Notably, evaluation of the performance of PGS_HL_ and PGS_CIO_ using summary statistics from the PCL cohort revealed that PGS_CIO_ (*P* = 5.54 × 10^− 5^) outperforms PGS_HL_ (*P* = 2.93 × 10^− 3^) in predicting CIO risk. This observation was replicated in the SJMB cohort, where PGS_CIO_ was significantly associated with CIO (*P* = 0.043), while PGS_HL_ was not (*P =* 0.52).

### Uncovering cells and pathways of relevance to CIO

While initial evaluations of the PGSs using summary statistics from the PCL cohort provided valuable insights, further validation of PGS_CIO_ using individual-level genotype and clinical data was essential to confirm the reliability of these findings. Subsequent analyses revealed that while the PGS_CIO_ was significantly associated with CIO in the SJMB cohort (*P* = 0.043), the inclusion of this score in a logistic regression model resulted in only a marginal improvement in predictive performance (AUC = 0.732) when compared to a model built with existing clinical/demographic predictors (age at diagnosis, CSI, protocol, and ancestral principal components) (AUC = 0.714). Similarly, while PGS_CIO_ accounted for more of the variance observed for CIO (Nagelkerke *R*^2^ = 0.024), compared to PGS_HL_ (Nagelkerke *R*^2^ = 0.006), highlighting the value of our filtering approach, the variance explained was still relatively low. Consequently, the current clinical utility of this score remains limited. Nonetheless, by integrating human CIO GWAS data with murine snRNA-seq data, we have gained valuable insights into potential biological mechanisms underlying CIO.

Analysis of the snRNAseq data generated by this study revealed that cisplatin treatment led to a decrease in the abundance of several cell types within the inner ear, as well as an increase in the abundance of certain clusters of macrophages. The identification of cochlear cells that exhibit differential abundance in the snRNA-seq data, including specialized auditory cells (spindle cells, OHCs and type I neurons), supporting cells (supporting cells, supporting structures 1, supporting structures 2 and tympanic border cells), immune cells (macrophages), and bone cells (osteoblasts and osteocytes), allowed us to pinpoint specific cell types that are impacted by cisplatin and may therefore be important in CIO. The stria vascularis, organ of Corti, and spiral ganglion have previously been implicated in CIO [28], with spindle cells, OHCs, and type I neurons residing in these respective structures. Although the role of supporting cells in CIO remains poorly understood, the observed decrease in abundance of these cells post-cisplatin treatment highlights the need to further investigate how these cells contribute to the development of CIO. In addition, it has been previously reported that cisplatin triggers inflammatory pathways, leading to the release of pro-inflammatory cytokines and the recruitment of immune cells such as macrophages [12], which is reflected by the observed increased abundance of macrophages post-cisplatin treatment. This inflammation can cause tissue damage and hearing loss [12] by compromising the structures responsible for auditory function. Macrophages, which are widely distributed throughout the cochlea, play a significant role in mediating this inflammatory response [14]. Lastly, cisplatin binds extensively to type I collagen in bone, creating a reservoir that gradually releases platinum, potentially leading to ototoxic effects [28]. By elucidating the contributions of these specific cochlear cells, our study significantly advances the understanding of CIO and offers novel insights into its underlying mechanisms, opening future avenues of research.

### Study limitations

While our study has yielded valuable insights relating to the genetics underlying CIO, it is important to acknowledge several limitations which may contribute to the limited clinical utility of this score. First, given the potential incomplete overlap in the underlying genetic architecture between hearing loss and CIO, hearing loss GWAS may not capture all genetic factors that contribute to CIO risk. Although the PGS_HL_ exhibited a significant association with CIO in the PCL cohort, the use of a future large-scale CIO GWAS as the base cohort for PGS development could provide deeper insights into the genetic determinants of CIO and enhance the accuracy and clinical applicability of this score. Further, many of the variants included in the initial PGS_HL_ mapped to non-coding regions. Therefore, future studies should explore the integration of single nuclei multi-omics data, which includes single-nuclei assay for transposase-accessible chromatin sequencing (snATAC-seq) data, to allow for the annotation and inclusion of intergenic variants in the biologically informed PGS. Our study also recognizes that while the genomes of humans and mice are genetically similar, with approximately 90% conserved regions [44] and an 85% overlap in protein-coding regions [45], they are not identical, and the importance of age-related differences between mouse and human samples should also be acknowledged. Future studies should, therefore, investigate gene expression changes in older murine models, which align more closely with the age demographics of our human samples.

Importantly, the data used in this study included individuals who were predominantly of European descent. Consequently, it is unknown whether the results from this study are transferable across global populations. Therefore, it is crucial to include individuals with non-European ancestries in future studies. Recognizing this limitation, current initiatives such as the All of Us Program and Our Future Health are focusing on assembling larger and more diverse biobanks [46]. Using these diverse datasets in future research will enhance our ability to ensure that PGSs are applicable to a broader range of individuals. Finally, inherent differences in patient demographics and variations in the definition and measurement of CIO between the PCL and SJMB cohorts can have unknown consequences for PGS_CIO_ [47]. The PCL cohort consists of childhood cancer survivors treated with or without carboplatin, alongside cisplatin, while the SJMB cohort focuses on medulloblastoma patients treated with cisplatin and CSI. Additionally, the PCL and SJMB cohorts employed different classification systems for CIO: the Muenster and Chang grading scales, respectively. This may lead to inconsistent classification of CIO cases, potentially impacting comparisons of PGS_CIO_ performance across these cohorts.

## Conclusion

Our study holds significant implications for understanding the genetics underlying CIO. By demonstrating the feasibility of integrating large-scale datasets, such as the hearing loss MTAG summary statistics data from the UKB cohort, with omics information, such as murine inner ear snRNA-seq data, we provide a novel approach for developing pharmacogenomic PGSs. Further, our identification of specific cochlear cell types involved in CIO sheds light on the intricate cellular pathways and processes underlying this ADR. This novel information will lay the groundwork for future research aimed at developing targeted therapeutic interventions to mitigate CIO risk. Overall, our study underscores the importance of interdisciplinary approaches to unravel the complexities of CIO and contributes to the growing body of literature that is improving our understanding of the etiology of this ADR.

## Electronic supplementary material

Below is the link to the electronic supplementary material.

Supplementary Material 1

## Data Availability

Data used in this study: MTAG summary statistics data for the base cohort was retrieved from the NeMo Archive (https://data.nemoarchive.org/other/grant/sament/sament/hearing_gwas/) on 26 March 2020. The summary statistics from the CIO GWAS were downloaded from the EBI GWAS Catalog (https://www.ebi.ac.uk/gwas/publications/34262104#study_panel) on 18 May 2021. The LD reference panel for SBayesR was downloaded from https://zenodo.org/record/3375373#.YYQZEL3MLfa on 7 June 2022.

## Code

Scripts that were used in this study are available on https://github.com/Drogemoller-Lab/Using-Genomics-to-Predict-and-Prevent-Cisplatin-Induced-Otoxicity, https://github.com/Drogemoller-Lab/CIO-PGS, and https://github.com/Drogemoller-Lab/scRNA-pipeline-UofM.
