# Supplementary material for: Leveraging large-scale datasets and single cell omics data to develop a polygenic score for cisplatin-induced ototoxicity
Source: Hum Genomics. 2024 Oct 8;18:112. doi: 10.1186/s40246-024-00679-5 (PMC11463131; doi:10.1186/s40246-024-00679-5)
Supplement: Supplementary file 1 — Supplementary Material 1 [file 40246_2024_679_MOESM1_ESM.pdf]

Supplementary Figures

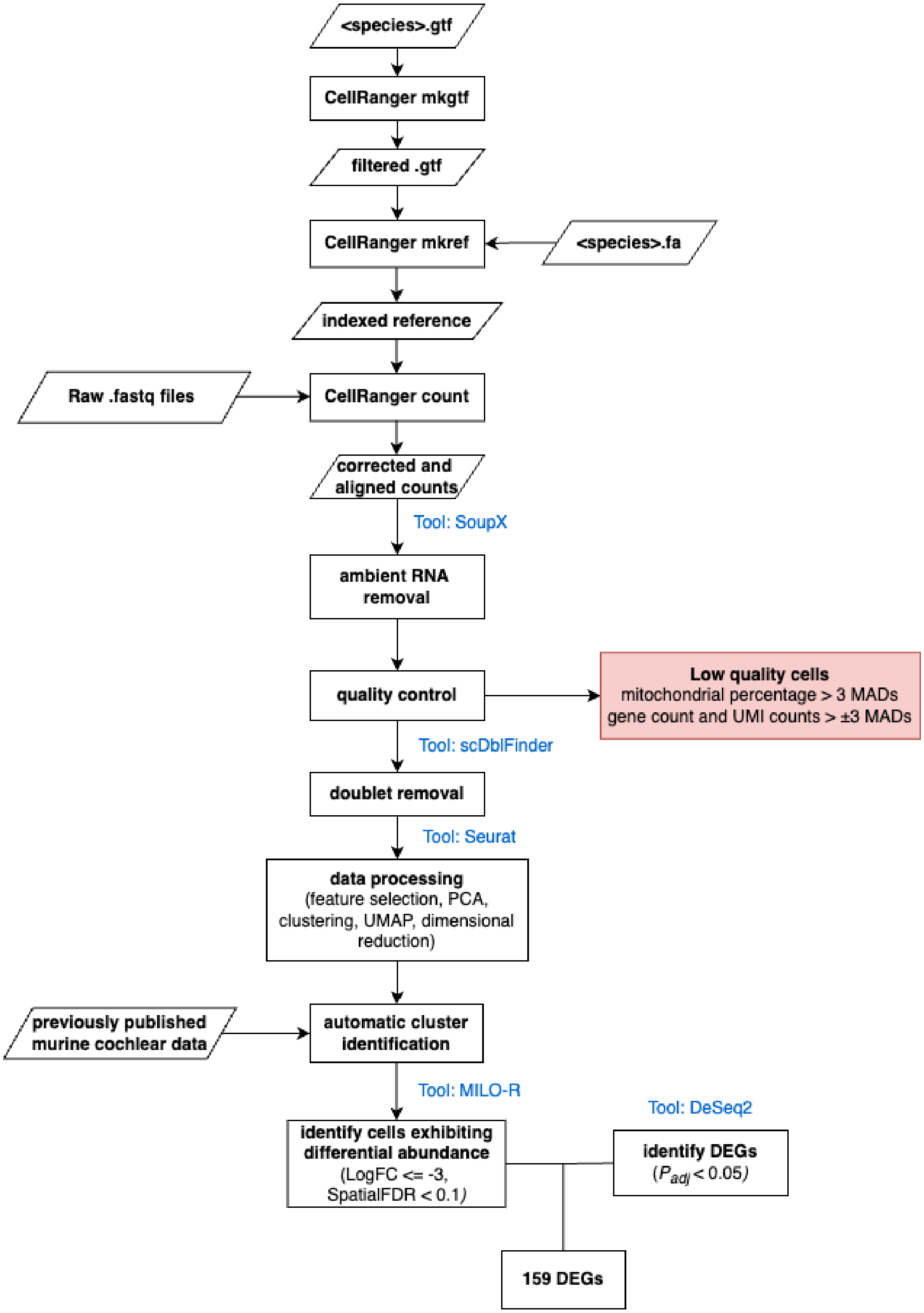

Supplementary Figure 1. Workflow summary of snRNA-seq analysis. PCA: principal component analyses, UMAP: uniform manifold and approximation projection, DEGs: differentially expressed genes.

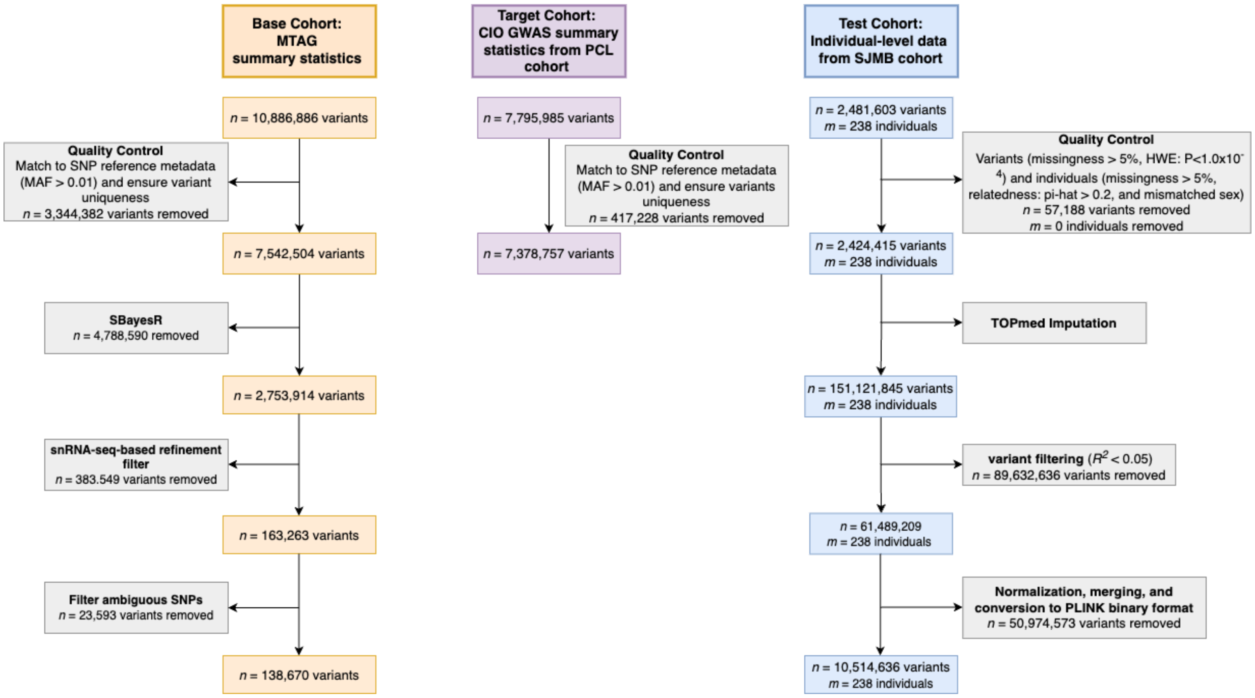

Supplementary Figure 2. Data processing of summary statistics (base and target cohorts) and individual-level data (test cohort). The gray boxes outline the steps and parameters used to process the data, including the number of variants and individuals that were filtered out. MTAG: multi-trait meta-analysis genome-wide association study, snRNA-seq: single-nuclei RNA sequencing, CIO: cisplatin-induced ototoxicity, GWAS: genome-wide association study, PCL: PanCareLIFE, SJMB: St. Jude Children's Research Hospital medulloblastoma.

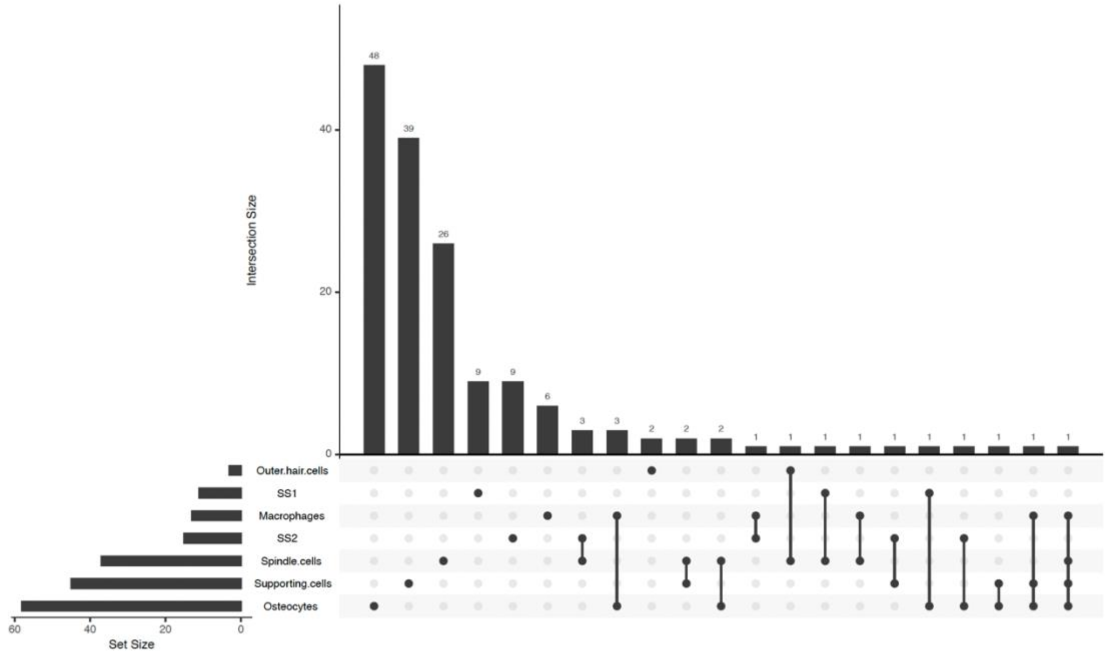

Supplementary Figure 3. UpSet plot of DEGs, which shows the total number of DEGs per cochlear cell type and the number of shared DEGs within cochlear cells. DEG: Differentially expressed genes. SS1: supporting structures 1; SS2: supporting structures 2.

a) b)

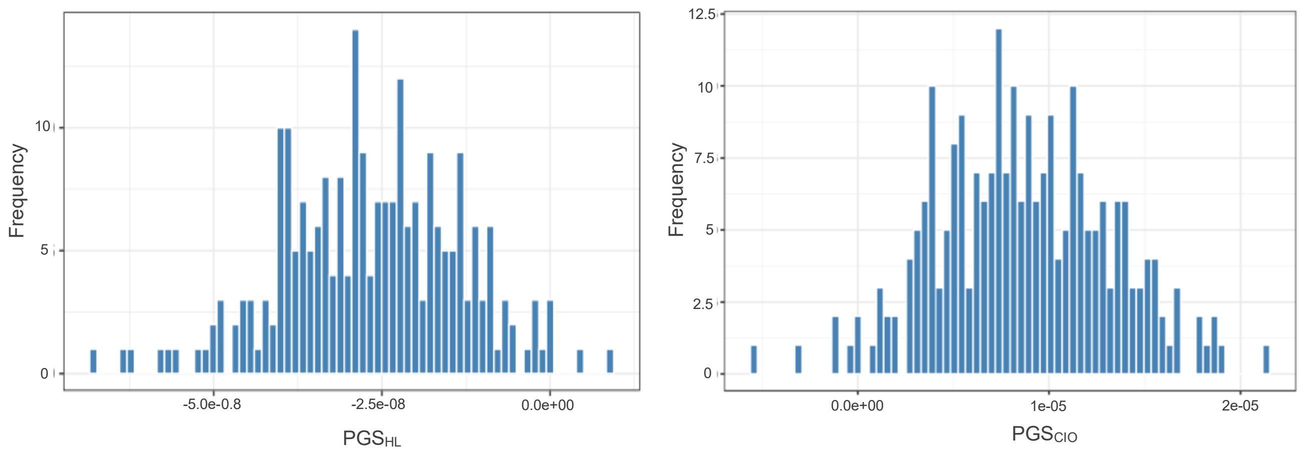

Supplementary Figure 4. Distribution of PGS_{HL} and PGS_{CIO} in the test cohort. Normality was assessed using the Shapiro-Wilk normality test (PGS_{HL}: P=0.72; PGS_{CIO}: P=0.96).

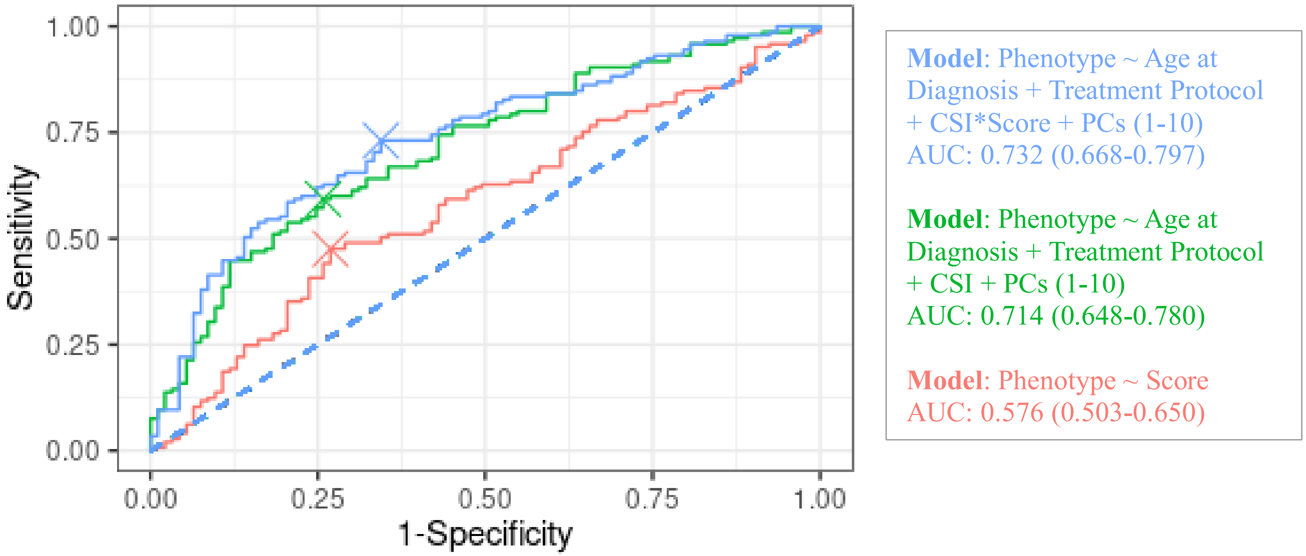

Supplementary Figure 5. Comparison of different logistic regression models. The models are PGS$_{\mathrm{CIO}}$ (red), clinical predictors only (green), and PGS$_{\mathrm{CIO}}$ + clinical predictors (blue). Integration of PGS$_{\mathrm{CIO}}$ into the model resulted in a slight improvement in predictive accuracy (AUC = 0.732).
